# Small Intestinal Bacterial Overgrowths and Intestinal Methanogen Overgrowths Breath Testing in a Real-Life French Cohort

**DOI:** 10.14309/ctg.0000000000000556

**Published:** 2022-12-08

**Authors:** Anne Plauzolles, Stella Uras, Guillaume Pénaranda, Marion Bonnet, Patrick Dukan, Frédérique Retornaz, Philippe Halfon

**Affiliations:** 1 Clinical Research and R&D Department, Laboratoire Européen Alphabio Biogroup, Marseille, France;; 2 Faculty of Sciences, Aix-Marseille University, Marseille, France;; 3 Infectious and Internal Medicine Department, Hôpital Européen Marseille, Marseille, France.

**Keywords:** small intestinal bacterial overgrowth, intestinal methanogen overgrowth, lactulose breath test, breath tracker

## Abstract

Breath testing has become a widely used tool to diagnose small intestinal bacterial overgrowths (SIBOs) and intestinal methanogen overgrowths (IMOs) in clinical settings. Owing to the heterogeneity in clinical manifestations and lack of standardization among centers performing breath testing, SIBO and IMO can be easily overlooked by the clinician. We studied the prevalence and symptoms of SIBO/IMO in French patients referred for breath testing after seeking medical advice.

**METHODS:**

Breath test data and symptoms of 331 patients were assessed for SIBO/IMO using the H_2_/CH_4_ lactulose breath test (LBT). Wilcoxon test or χ^2^ test were used to compare patients with SIBO/IMO with patients without SIBO/IMO. LBT positive patients (H_2_+, CH_4_+, and CH_4_+/H_2_+) were compared using Kruskal-Wallis test for continuous data or χ^2^ test for categorical data.

**RESULTS:**

Among the 186 (68.1%) patients tested positive for an overgrowth with 40.3%, 47.3%, and 12.4% for H_2_+, CH_4_+ and CH_4_+/H_2_+, respectively, the presence of diarrhea was significantly increased in hydrogen type overgrowths (*P* < 0.001). No significant difference according to age, gender, and symptoms was associated with a positive test except for joint pain that was less prevalent among LBT positive patients (*P* = 0.038). In 86.5% of IMOs, positivity with CH_4_ values ≥10 ppm could be identified at baseline.

**DISCUSSION:**

There are little discriminating symptoms that can help the clinician to identify patients likely to have a SIBO/IMO. However, SIBO/IMOs remain a common disorder widely underdiagnosed that need further studies to better apprehend functional bowel disorders.

## INTRODUCTION

In the past decades, breath testing has become a commonly noninvasive tool to diagnose the pathologic bacterial fermentation within the small bowel also referred as SIBO for small intestinal bacterial overgrowth (1–5). The key concept of breath test relies on the production of hydrogen (H_2_) by colonic bacterial fermentation which then diffuses into the blood and is excreted by breath where it can be measured (6). In parallel, other gases are today routinely measured including methane (CH_4_) for the detection of intestinal methanogen overgrowth (IMO, also previously referred as SIBO CH_4_) and most recently hydrogen sulphide (H_2_S) for the detection of SIBO H_2_S. Because human cells do not produce these gases, they can be measured in clinical breath testing for the detection of abnormal microbial overgrowths in the digestive tract (7).

The signs and symptoms associated with SIBO/IMOs are nonspecific. Hence, numerous digestive and extradigestive symptoms have been linked to SIBO/IMOs, but they cannot be used in isolation to diagnose an abnormal overgrowth of microbes. Among the numerous symptoms overgrowths have been linked to, gut dysbiosis including SIBO and IMO generally causes irritable bowel syndrome-like symptoms with abdominal pain, distention, altered bowel habits (diarrhea and/or constipation), and bloating (8,9). Severe manifestations of SIBO/IMO are rare and not systematic, they comprise nutritional deficiencies, weight loss, and anemia. Overall, most SIBO/IMO patients are either clinically asymptomatic or have symptoms that can overlap with other gastrointestinal disorders (10,11). Accordingly, SIBO/IMOs have been linked to numerous diseases (12–20) which usually affect either 1 or both intestinal secretion and/or motility and physiological protective mechanisms regulating the colonization of our gut by microorganisms. By opposition to the previous invasive investigation (endoscopy and jejunal fluid aspiration for bacterial cultures), breath tests are nowadays relatively simple and safe to perform on adults and children; however, the heterogeneous nature of SIBO/IMOs makes it a difficult-to-diagnose condition which is currently largely underdiagnosed. It is important for clinicians to understand the health conditions and risk factors that predispose to the development of these abnormal overgrowths. Today, practitioners are challenged by the lack of a scientifically validated diagnostic gold standard and well-designed clinical trials for the management of SIBO/IMO.

When it comes to diagnosing and treating SIBO/IMOs, the ambiguity surrounding this disorder is compounded by the lack of consensus. In 2017, a consensus was reached by a group of experts who were selected based on a proven knowledge and/or expertise in breath testing (4). This consensus also referred as the North American Consensus (NAC) aimed at providing guidelines for physicians utilizing breath testing in their clinical practice. The NAC comes as an update of the 2 previous consensuses published in 2009 (2) and 2005 (3) taking into consideration the recent evolution of knowledge accumulated by the scientific community in the field of the microbiome. Each NAC statement was voted by experts and classified from high to very low as a quality of evidence. Full agreement was not reached on each topic discussed thus emphasizing the difficulty to establish a common agreement on indications for breath testing, the preparation and performance of the test, and the interpretation of the results. Most recently, a European guideline (5) was published, but there are still some gaps uncovered including the detection and interpretation of H_2_S, which has only recently become available in routine testing. Furthermore, the understanding of the gas exchange dynamic seems essential in the interpretation of breath testing (21). Updates of the existing consensuses and guidelines arise in the literature (22–25), but standardization is lacking regarding indications for testing, test methodology, and interpretation because this field is constantly evolving. Practitioners are in need for an evidence-based approach for the management of SIBO/IMO, including well-designed clinical trials for its treatments.

We evaluated the prevalence and the symptoms of SIBO/IMO in a real-life population who underwent breath testing in a French laboratory after medical advice.

## METHODS

### Study design

We retrospectively reviewed the results of lactulose breath tests (LBTs) performed in a French laboratory from November 2020 to June 2021. Before testing, all patients (n = 331) completed a self-report questionnaire. The questionnaire involved rating symptoms known to be potentially linked or associated to overgrowths including digestive and extradigestive symptoms. Symptoms had to be self-reported only if they were chronic or recurrent at least in the last 3 months. Bowel movements with Bristol stool types were collected. If the patient presented alternating bowel movements and Bristol stool types, he/she could also indicate his/her bowel habit during acute constipation/diarrhea. Constipation was defined as dry and hardened stools 3 or fewer times per week, whereas diarrhea was defined as watery stools 3 or more times a day. Self-reported questionnaires and adherence to the preparation diet were systematically checked by a physician before starting breath testing. Patients who did not complete the test (interruption before the 180 minute measure) or did not comply to the preparation guidelines for the examination were excluded. Briefly, patients could not perform the test if they had taken antibiotics in the preceding month before the examination (except patients treating a SIBO/IMO previously diagnosed), used laxative or prokinetics at least 7 days before the examination, patients who had smoked 12 hours before the test, and patients who did not adhere to the breath test preparation diet the day before the examination. Patients were not tested if they had undergone a bowel preparation for colonoscopy within 4 weeks preceding the examination.

### Data analysis

A retrospective analysis of breath test data from patients evaluated for microbial overgrowth over the study period was performed. A baseline breath sample was obtained; a 10 g oral lactulose load was given; alveolar breath samples were obtained at 20-minute intervals for 3 hours and analyzed with a Gastrogenius Breath tracker (Laborie). The breath-tracker machine works by checking the breath sample for an appropriate amount of oxygen (O_2_) and the H_2_ and CH_4_ present. The machine uses an O_2_ correction factor technique to minimize errors caused by improper sampling techniques. If the sample does have too much O_2_, it was considered invalid. When the measure was invalid, the patient was asked to provide immediately another sample. H_2_ and CH_4_ measures were interpreted using published criteria (4). For H_2_, an increase in exhaled hydrogen of at least 20 parts per million (ppm) above baseline within 90 minutes of oral ingestion of lactulose was interpreted as abnormal (positive for H_2_). For CH_4_, any value of 10 and above within 180 minutes of oral ingestion of the substrate was considered abnormal (positive for CH_4_). Breath test data that did not meet these criteria were deemed SIBO/IMO negative. If elevated baselines for H_2_ (>19 ppm) and/or CH_4_ (>9 ppm) were measured, the physician checked a second time the patient's compliance to the test guidelines and adherence to the preparation diet detailing food intake for the past 24–48 hours as well as treatments and medical testings the patient may have undertaken over the past month. Elevated baselines were defined as positive when good compliance with the test guidelines and preparation diet were validated. Flat lines throughout the 180 minute of testing (H_2_ < 10 ppm and CH_4_ < 3 ppm) were excluded from this analysis because these values could lead to different conclusions, suggesting either a negative SIBO/IMO test or the presence of a potential SIBO H_2_S.

### Statistical analysis

Data were reported using mean (SD) or median (Q1–Q3) according to the type of variables. First, Wilcoxon test or χ^2^ test were used to compare patients with SIBO/IMO with patients without SIBO/IMO. Second, SIBO/IMO patients were divided into 3 groups (H_2_+ SIBO, CH_4_+ SIBO, and CH_4_+/H_2_+ SIBO) according to the results of breath test. Groups were compared using Kruskal-Wallis test for continuous data or χ^2^ test for categorical data. If a test showed a significant *P* value, post hoc comparisons where performed. *P* values were adjusted with the Bonferroni method accounting for multiple comparisons. Positive breath test patterns were compared using χ^2^ test or Fisher test. Statistical significance was set to α < 0.05. Statistical analyses were performed using R-Studio (R-Studio, version 1.4.1106, 2009–2021, PBC).

### Ethical issues

The results were obtained from a French clinical laboratory (Alphabio Biogroup Laboratory, Marseille, France). According to French regulations, the study was approved by French ethics committees (CPP Sud-Méditerranée II and Health Data Hub, approval number: F20211014100510: https://www.health-data-hub.fr/projets/etude-par-test-respiratoire-de-la-prevalence-des-pullulations-microbiennes-siboimo-et-leurs). All data were fully anonymized before analyses.

## RESULTS

### Demographic and clinical characteristics of the cohort

**Figure 1.**
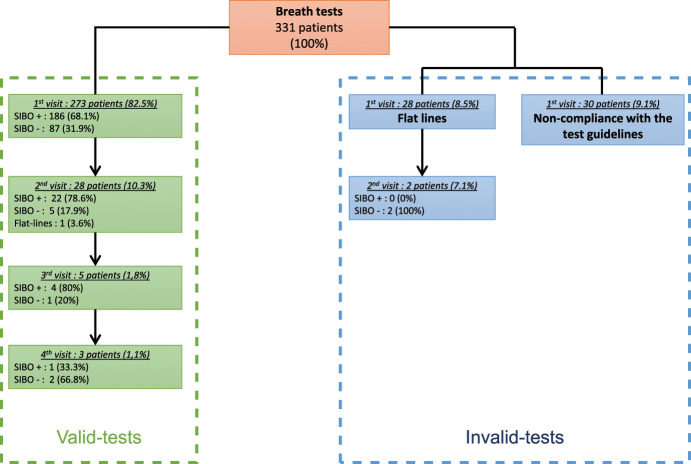
Flow chart of the breath tests validity in this real-life cohort. This figure illustrates the breath test selection of the 331 patients who underwent a breath test at a French clinical laboratory (Alphabio Biogroup Laboratory, Marseille, France) between November 2020 and June 2021. Two hundred seventy-three patients with a total of 309 breath test records were selected and interpreted based on Rezaie et al. (39), the remaining 58 patients were excluded from this study as invalid tests as they could not provide reliable breath test results.

Among the 331 patients (Figure 1) who came to the laboratory during the study period to perform a LBT, 30 (9.1%) subjects were not included in the study because they did not comply to the test guidelines: 9 had not followed the restriction diet, 4 had recently used antibiotics, 4 were unable to provide a valid sample, 3 had smoked less than 12 hours before the test, 1 could not drink the whole lactulose load, 1 used laxative the day before the test, and 8 medical records only indicate a noncompliance with the test guidelines. In addition, 28 (8.5%) tests showing flat lines were excluded from this analysis because data could lead to erroneous diagnosis with H_2_ and CH_4_ values suggesting either a negative result or a suspicion of SIBO H_2_S. Therefore, data of 273 patients (median age 45 [range 13–87]), 205 women (75.1%) and 68 men (24.9%) were analyzed (Table 1). Patients enrolled in this study were referred to our laboratory after seeking medical advice; they presented with an average of 9.3 (SD 3.6) symptoms among the symptoms listed in the questionnaire with most digestive symptoms (5.8/10 on average, SD 2.2) compared with extradigestive symptoms (3.5/7 on average, SD 1.9). The most prevalent symptoms (>70%) being flatulence (86.8%), bloating and distension (83.9%), abdominal pain (76.9%), and fatigue (74.4%). Age and gender did not differ significantly between SIBO/IMO positive and SIBO/IMO negative groups. The overall symptoms of the patients studied were equally distributed between SIBO/IMO positive and SIBO/IMO negative patients as displayed, except for joint pain, which was more common in patients with a negative LBT. Further analysis looking at the influence of age on the prevalence of joint pain did not show age as a confounding factor (see Appendix 1, Supplementary Digital Content, http://links.lww.com/CTG/A898).

**Table 1.** Demographic and clinical characteristics of the cohort

Characteristic n (%)	Patients^a^ 273 (100)	SIBO/IMO− 87 (31.9)	SIBO/IMO+ 186 (68.1)	*P* value
Age (yr)^b^	45 (13–87)	46 (13–75)	44 (20–87)	0.415
Age, n (%)				
0–40	107 (39.2)	32 (36.7)	75 (40.3)	0.822
40–60	120 (44)	39 (44.8)	81 (43.6)	
>60	46 (16.9)	16 (18.4)	30 (16.1)	
Sex, n (%)				
Female	205 (75.1)	68 (78.2)	137 (72.1)	0.515
Male	68 (24.9)	19 (21.8)	49 (26.3)	
Symptoms (more than 1 symptom possible)^c^				
Digestive, n (%)				
Abdominal pain	210 (76.9)	67 (77)	143 (76.9)	0.999
Bloating	229 (83.9)	74 (85.1)	155 (83.3)	0.854
Constipation	141 (51.7)	43 (49.4)	98 (52.7)	0.709
Diarrhea	137 (50.2)	47 (54)	90 (48.4)	0.461
Flatulence, gas	237 (86.8)	74 (88.1)	163 (87.6)	0.693
Food intolerance	109 (39.9)	37 (42.5)	72 (38.7)	0.640
Indigestion	169 (61.9)	54 (62.1)	115 (61.8)	0.999
Nausea	104 (38.1)	37 (43.5)	67 (36)	0.369
Transit alteration	149 (54.6)	50 (57.5)	99 (53.2)	0.599
Extradigestive, n (%)				
Anxiety	119 (43.6)	43 (49.4)	76 (40.9)	0.231
Brain fog	123 (45.1)	43 (49.4)	80 (43)	0.389
Dizziness	107 (39.2)	35 (40.2)	72 (38.7)	0.915
Fatigue	203 (74.4)	65 (32)	138 (74.2)	0.999
Headaches	132 (48.4)	47 (54)	85 (45.7)	0.249
Heartburns	134 (49.1)	49 (56.3)	85 (45.7)	0.132
Joint pain	121 (44.3)	47 (54)	74 (39.8)	0.038
Skin disorders	104 (38.1)	34 (39.1)	70 (37.6)	0.924

F, female; IMO, intestinal methanogen overgrowth; M, male; SIBO, small intestinal bacterial overgrowth; SIBO+, patient with a breath test positive; SIBO−, patients with a breath test negative.

a Breath tests performed as follow-up for patients previously diagnosed and treated for their SIBO/IMO are excluded from this table.

b Median (min–max).

c Symptoms collected at first visit.

### Results of breath tests

A total of 68.1% (186) patients were LBT positive including 98 (52.7%) H_2_ positive and 111 (59.7%) CH_4_ positive. Of those, 75 (40.3%) were H_2_ positive alone, 88 (47.3%) were CH_4_ positive alone, and 23 (12.4%) were dual-positive for H_2_ and CH_4_ (Table 2). The median age of the dual positive group is smaller than the 1-type overgrowths (*P* = 0.032). No statistically significant difference was seen in constipation or diarrhea between patients with a normal LBT vs those with a positive LBT. However, for the patients who tested positive, diarrhea was significantly increased in hydrogen type overgrowths (H_2_+ with or without CH_4_+) (*P* < 0.001), whereas constipation seemed more prevalent in methane type overgrowths (CH_4_+ with or without H_2_+) but did not reach statistical significance (*P* = 0.079). Of the 111 methane positive tests (CH_4_+ with or without H_2_+), 96 (86.5%) had a baseline displaying a positive CH_4_ LBT with level ≥10 ppm before the ingestion of lactulose. No association was established between the prevalence of constipation and/or diarrhea, and the ppm values measured whether they were high or low (*P* = 0.562 for constipation and *P* = 0.345 for diarrhea) (Table 3).

**Table 2.** Positive hydrogen and methane breath tests at first visit

Characteristic n (%)	CH_4_+ 88 (47.3)	CH_4_+/H_2_+ 23 (12.4)	H_2_+ 75 (40.3)	*P* value
Age (yr)^a^	48 (20–87)	34 (22–65)	43 (22–70)	0.032
Age, n (%)				
0–40	31 (35.2)	14 (60.9)	30 (40)	0.162
40–60	39 (44.3)	8 (34.8)	34 (45.3)	
>60	18 (20.5)	1 (4.4)	11 (14.7)	
Sex, n (%)				
Female	67 (76.1)	18 (78.3)	52 (69.3)	0.535
Male	21 (23.9)	5 (21.7)	23 (30.7)	
Symptoms (more than 1 symptom possible), n (%)				
Abdominal pain	66 (75)	17 (73.9)	60 (80)	0.705
Anxiety	38 (43.2)	8 (34.8)	30 (40)	0.752
Bloating	74 (84.1)	18 (78.3)	63 (84)	0.784
Brain fog	35 (39.8)	9 (39.1)	36 (48)	0.527
Constipation	52 (59.1)	14 (60.9)	32 (42.7)	0.079
Diarrhea	28 (31.8)	12 (52.2)	50 (66.7)	<0.001
Dizziness	36 (40.9)	8 (34.8)	28 (37.3)	0.823
Fatigue	64 (72.7)	16 (69.6)	58 (77.3)	0.690
Flatulence, gas	78 (88.6)	17 (73.9)	68 (90.7)	0.095
Food intolerances	32 (36.4)	6 (26.1)	34 (45.3)	0.208
Headaches	41 (46.6)	7 (30.4)	37 (49.3)	0.274
Heartburns	41 (46.6)	7 (30.4)	37 (49.3)	0.274
Indigestion	55 (62.5)	10 (43.5)	50 (66.7)	0.133
Joint pain	31 (35.2)	8 (34.8)	35 (46.7)	0.289
Nausea	32 (36.4)	8 (34.8)	27 (36)	0.990
Transit alteration	46 (52.3)	11 (47.8)	42 (56)	0.766
Skin disorders	34 (38.6)	8 (34.8)	28 (37.3)	0.942
No. of symptoms^a^				
Digestif	6 (0–10)	5 (1–8)	6 (1–10)	0.201^b^
Extradigestive	3 (0–7)	2 (0–6)	3 (0–7)	0.457^b^

CH_4_+, patient with a breath test positive for an IMO; CH_4_+/H_2_+, patient with a dual-positive breath test for a SIBO and IMO; H_2_+, patients with a breath test positive for a SIBO; IMO, intestinal methanogen overgrowth; SIBO, small intestinal bacterial overgrowth.

a Median (min–max).

b Kruskal-Wallis test.

**Table 3.** Positive breath test patterns correlated to the presence or absence of diarrhea and constipation in positive breath tests

Breath test measures n (%)	Diarrhea (−) 25 (33.3)	Diarrhea (+) 50 (66.7)	*P* value
H_2_ (ppm)			0.345
Elevated baseline (T0 >20 ppm)	8 (32)	11 (22)	
Increase 20–40 ppm between T0 and T90 min	9 (36)	20 (40)	
Increase 40–80 ppm between T0 and T90 min	8 (32)	14 (28)	
Increase >80 ppm between T0 and T90 min	0 (0)	5 (10)	

	Constipation (−) 45 (40.5)	Constipation (+) 66 (59.5)	
CH_4_ (ppm), peak value (T0-T180 min)			0.562
10–40 ppm	23 (51.1)	28 (42.4)	
40–80 ppm	14 (31.1)	27 (40.9)	
>80 ppm	8 (17.8)	11 (16.7)	
CH_4_ elevated baseline (T0)	38 (84.4)	58 (87.9)	0.7782

ppm, parts per million.

Of the 331 patients, 30 (9.1%) came for testing several times over the study period. Five patients came at least 3 times of which 2/5 (40%) turned their SIBO breath test negative throughout the following testing (see Appendix 2, Supplementary Digital Content, http://links.lww.com/CTG/A898).

## DISCUSSION

Because patients with SIBO/IMO may be clinically asymptomatic or have symptoms that can overlap with other gastrointestinal disorders, identifying patients likely to be good candidates for breath testing remains challenging for clinicians. This study was conducted in a real-life French cohort of patients with symptoms of unclear cause of which most presented with gut disorders. Our results show that no specific symptoms could discriminate the patients who presented a positive LBT vs the one who had a negative LBT. However, diarrhea was significantly increased with H_2_-type overgrowth as opposite to the CH_4_-type overgrowth. Furthermore, up to 86.5% of patients diagnosed with a methane-type overgrowth (using NAC guidelines as reference standard) presented an elevated baseline that was sufficient to identify the IMO before the ingestion of lactulose.

Several articles have shown a greater prevalence of SIBO/IMOs according to certain physiological characteristics and other factors such as age (26), sex (26), PPI, or anxiolytic intakes (27–31), but these associations are not demonstrated in all studies, which is also the case in our cohort. There is a wide range of symptoms that have been associated to intestinal abnormal overgrowths in the literature, but the various prevalence of symptoms including the most common (bloating, flatulence, abdominal pain/discomfort, and altered bowel habits) show that signs and symptoms associated with SIBO/IMOs are nonspecific (11,19,32). This real-life study included mainly patients seeking medical advice who presented similar symptoms regardless of their SIBO/IMO status. Interestingly, once the overgrowth is established, transit alterations including constipation and diarrhea seem to be discriminants with a significant increase of diarrhea among patients with hydrogen-type overgrowths, whereas constipation seems more prevalent with methane-type overgrowths but did not reach statistical significance. Although no clear explanation has yet been suggested for the correlation H_2_-type SIBO and a higher prevalence of diarrhea, methane on the contrary is known to affect ileal and colonic transit times which favor intestinal stasis and constipation (33–36).

In our dataset, a single fasting measurement seemed sufficient to diagnose a methane overgrowth. It has been suggested in the literature that breath methane can nearly always be detected at baseline (37,38). Hence, patients with excessive methane continue to excrete high levels of methane in the fasting state thus resulting in subsequent increases more modest than with hydrogen as time passes when undertaking a breath test. Rezaie et al. (39) in 2015 and most recently Takakura et al. (40) in 2022 addressed this question and showed that a single fasting measurement of exhaled methane could be highly sensitive (96.1% and 86.4%, respectively) and specific (99.7% and 100%, respectively) in identifying excessive methane producers compared with full lactulose breath testing. With a sensitivity of 86.5% and a specificity of 98.8%, our real-life data here correlate almost perfectly with recent double-blind randomized control trial by Takakura. These data comfort the idea that a spot methane test may be sufficient for the detection of most IMOs and could be substituted for the full breath test for patients with an elevated CH_4_ baseline. This approach could present the advantages to shorten the test and avoid the patient to go through symptoms that may occur after the ingestion of lactulose. Furthermore, when analyzing the range of ppm values and the prevalence of transit alterations (constipation and diarrhea) in LBT positive patients, no significant correlation appeared in our dataset, suggesting that patients may show different sensitivities toward the presence of gases in their gut, especially with methane that is known to affect gut motility. These findings further accentuate the already existing debates concerning the relevance of lowering the cut-off value for the detection of IMOs, one of the items that was previously discussed for the NAC but of which experts did not manage to reach an agreement.

In this cohort, we are looking at a population who is seeking for medical advice. Unlike study cohorts which are compared with healthy participants, the group of SIBO/IMO negative patients which refers as our control group here presents symptoms that may be because of other health conditions. However, the high prevalence of positive LBT (68.1%) in this cohort comforts the idea that SIBO/IMOs are largely underdiagnosed in the French population and the practice of breath testing should be implemented in routine testing. Overall, this cohort reveals that breath tests are mostly prescribed to patients experiencing gastrointestinal disorders; however, the presence of digestive symptoms does not seem sufficient to predict the presence of abnormal overgrowths in the digestive tract. Although many studies often confront SIBO/IMO positive patients vs healthy individuals, some focus on a specific pathology to define the prevalence of SIBO/IMO among the patients but little declare discriminating symptoms among symptomatologic patients based on their SIBO/IMO status either positive or negative. Hence, the sole evaluation of symptoms does not seem sufficient for the clinician to recommend that the patient undergo a breath test. In the literature, questionnaires or differential diagnostic can help target whether a patient could be positive for an overgrowth (41–43).

This study has several strengths including a significant number of patients with a wide range of symptoms and strict guidelines compliance accordingly to the latest guidelines at the time of the study. Being a real-life study, its main limitations relate to the lack of a healthy control group and is limited to the information collected to undertake the test thus excluding clinical information that could highlight SIBO/IMOs' predictors and characteristics. Linking the prevalence of SIBO/IMO to other health conditions could have been a potential indicator of SIBO/IMO in this cohort because studies show that some conditions are known to favor abnormal overgrowths (19,20,44,45). In this real-life cohort, we are limited to the information collected to undertake the test and could not correlate health condition concomitant with SIBOs and IMOs. Finally, the retrospective nature of this study is a limitation in clearly identifying the symptoms correlated to this disorder. Looking at a follow-up testing could have strengthened the association between SIBO prevalence and symptoms because true correlations would have been highlighted when symptoms improve or disappear with overgrowth's eradication, but too little follow-up tests were performed over the studied period.

In practice, the heterogenous nature of SIBO/IMO manifestations in breath test positive patients might be confounded with numerous other coexisting health conditions thus making it challenging for clinicians to identify the patients who need to be tested. Epidemiological data are missing in the literature because even healthy patients may have a positive breath test despite the absence of signs and symptoms (10). Hence, it seems that SIBO/IMO might be underestimated because of its nonspecific signs and symptoms and the lack of breath testing prescribed by clinicians thus emphasizing the need to implement routine breath testing to monitor SIBO/IMOs especially in patients with symptoms of unclear cause or when a clinician must deal with a difficult diagnosis. To do so, it is important for clinicians to understand the health conditions and risk factors that predispose to the development of SIBO/IMO. Although SIBO/IMO are now increasingly considered in clinician's differential diagnosis especially in difficult-to-diagnose patients with nonspecific gastrointestinal complaints, their clinical manifestations are complex and highly variable from one patient to another. Today, practitioners face controversies and inconsistencies in the interpretation of these tests and are challenged by the lack of a scientifically validated diagnostic gold standard and well-designed clinical trials for the management of SIBO/IMO. Further studies are needed as an increased awareness and understanding of these pathological overgrowths could help the clinicians and their patients without a unifying diagnosis.

## CONFLICTS OF INTEREST

**Guarantor of the article:** Anne Plauzolles, PhD.

**Specific author contributions:** A.P.: collected and analyzed data and drafted and finalized the manuscript. S.U. and G.P.: analyzed data. M.B., P.D., F.R., and P.H.: finalized the manuscript. All authors discussed the results and commented on the manuscript.

**Financial support:** None to report.

**Potential competing interests:** None to report.

## Supplementary Material

SUPPLEMENTARY MATERIAL

## Notes

Study Highlights WHAT IS KNOWN

✓ Small intestinal bacterial overgrowths (SIBOs)/intestinal methanogen overgrowth (IMOs) are highly prevalent in populations with gastrointestinal symptoms but remain largely underdiagnosed.
✓ Signs and symptoms associated with SIBO/IMOs are nonspecific and can overlap with concomitant health disorders.
✓ Standards for breath gas measurements in the detection of abnormal microbial overgrowths are evolving but robust guidelines are lacking.

WHAT IS NEW HERE

✓ There is a high prevalence (68.1%) of SIBO/IMOs in French patients presenting symptoms of unclear cause.
✓ The severity of signs and symptoms associated with SIBO/IMOs does not seems to correlate to the ppm values measured in exhaled breath testing.
✓ Up to 86.5% of methane positive patients were identified before ingestion of lactulose after a preparation diet.
