# Supplementary material for: Small Intestinal Bacterial Overgrowths and Intestinal Methanogen Overgrowths Breath Testing in a Real-Life French Cohort
Source: Clin Transl Gastroenterol. 2022 Dec 8;14(4):e00556. doi: 10.14309/ctg.0000000000000556 (PMC10132713; doi:10.14309/ctg.0000000000000556)
Supplement: SUPPLEMENTARY MATERIAL [file ct9-14-e00556-s001.docx]

**Supplemental material**

**Appendice 1: Influence of the age cohort on the prevalence of joint pain in regard to breath test results**

| Age n (%) | SIBO/IMO –  47 (54.7) | SIBO/IMO +  74 (39.6) | *p*-value |
| --- | --- | --- | --- |
| **Age (years)**  [0-40]  ]40-60]  >60 | 14 (29.8)  23 (48.9)  10 (21.3) | 23 (31.1)  38 (51.4)  13 (17.6) | 0.8790 |

**Appendice 1 (legend):** this table shows the prevalence of joint paint in patients with positive LBTs versus negative LBTs in correlation to age.

**Appendice 2: Cohort patients tested multiple times for overgrowth detection**

| Test 1 | Test 2 | Test 3 | Test 4 |
| --- | --- | --- | --- |
| CH4+  (14) | CH4^+^ (11) | CH4^+^ (3) | CH4^+^ (1) |
|  |  |  | NEGATIVE (1) |
|  | CH4^+^/H2^+^(2) | H2^+^ (1) |  |
|  | H2 (1) |  |  |
| H2+  (5) | H2^+^ (2) |  |  |
|  | Flat lines (1) |  |  |
|  | NEGATIVE (2) | NEGATIVE (1) | NEGATIVE (1) |
| CH4+/H2+  (2) | H2^+^ (1) |  |  |
|  | NEGATIVE (1) |  |  |
| NEGATIVE  (7) | NEGATIVE (2) |  |  |
|  | H2^+^ (2) |  |  |
|  | CH4^+^ (3) |  |  |
| Flat lines (2) | NEGATIVE (2) |  |  |

**Appendice 2 (legend):** this table shows breath test results of patients who underwent multiple breath testing over the study period.
